# Case Report: Sudden very late-onset near fatal PD1 inhibitor-associated myocarditis with out-of-hospital cardiac arrest after >2.5 years of pembrolizumab treatment

**DOI:** 10.3389/fcvm.2024.1328378

**Published:** 2024-02-19

**Authors:** Richard I. Lewis, Katharina Seuthe, Simon Lennartz, Jan-Phillip Weber, Nicole Kreuzberg, Karin Klingel, Paul J. Bröckelmann

**Affiliations:** ^1^ Department I of Internal Medicine, Center for Integrated Oncology Aachen Bonn Cologne Duesseldorf, Faculty of Medicine and University Hospital of Cologne, University of Cologne, Cologne, Germany; ^2^ Department III of Internal Medicine, Faculty of Medicine and University Hospital of Cologne, University of Cologne, Cologne, Germany; ^3^ Department of Radiology, Faculty of Medicine and University Hospital of Cologne, University of Cologne, Cologne, Germany; ^4^ Center for Hematology and Oncology, Oncology Cologne, Cologne, Germany; ^5^ Department of Dermatology, Faculty of Medicine and University Hospital of Cologne, University of Cologne, Cologne, Germany; ^6^ Cardiopathology, Institute for Pathology and Neuropathology, University Hospital Tübingen, Tübingen, Germany

**Keywords:** cancer immunotherapy, immune checkpoint inhibition, immune related adverse effects (irAEs), immune-related myocarditis, pembrolizumab

## Abstract

**Introduction:**

Immune checkpoint inhibitors have advanced the outcomes of many different types of cancer. A rare but extraordinarily severe complication of these agents resembles immune checkpoint inhibitor-related myocarditis, which typically occurs within the first few weeks after treatment initiation with a mortality of 25%–50%.

**Case report:**

A 57-year-old woman had uneventfully received pembrolizumab for metastatic non-small cell lung cancer for over 2.5 years and was admitted after an out-of-hospital cardiac arrest due to ventricular fibrillation. After successful cardiopulmonary resuscitation, the initial diagnostic work-up showed elevated cardiac enzymes and a limited left-ventricular ejection fraction, while coronary angiography did not show relevant stenosis. Despite cardiac MRI being unsuggestive of myocarditis, myocardial biopsies were obtained and histologically confirmed anti-PD-1 antibody-associated myocarditis. After the initiation of prednisone at 1 mg/kg body weight, the patient gradually recovered and was discharged three weeks later with markedly improved cardiac function.

**Conclusion:**

This case resembles the first description of a very late onset irMyocarditis, occurring over 2.5 years after the start of treatment. It demonstrates the importance of contemplating that severe immune-related toxicities with a sudden onset clinical presentation may occur even after long uneventful periods of anti-PD-1 immune checkpoint inhibitor treatment. Furthermore, it underlines the critical importance of myocardial biopsies in this setting, especially when cardiac MRI remains inconclusive. Moreover, it demonstrates the necessity and benefits of early immunosuppressive treatment if immune-related myocarditis is considered a differential diagnosis.

## Introduction

Since the discovery of immune checkpoint inhibition (ICI) as a novel cancer treatment paradigm, an ever-growing group of substances rapidly became part of standard treatments across major cancer types. For many, ICI revolutionized outcomes and is thus increasingly used in curative intent (1). With a reported incidence between 0.04% and 1.14%, ICI-related myocarditis (irMyocarditis) is a relatively rare, yet potentially lethal immune-related adverse effect (irAE) (2). Importantly, irMyocarditis is considered an early irAE, with a median occurrence of 17 to 34 days after treatment initiation, and 76% of cases develop within the first six weeks of ICI (3–5). However, in the following, we present a rare case of histologically confirmed near fatal irMyocarditis, which occurred after over 2.5 years of pembrolizumab treatment. To our knowledge, this resembles the longest yet reported latency of irMyocarditis.

## Case report

A 57-year-old woman experienced sudden cardiac arrest while attending a social event. After a no-flow time of approximately 1 min, a coincidentally present nurse initiated cardiopulmonary resuscitation (CPR). When paramedics arrived, approximately 10 min into CPR, the initial rhythm was ventricular fibrillation. The patient underwent a series of 3 defibrillations and received 200 mg of amiodarone before achieving a return of spontaneous circulation after a total of approximately 17 min.

**Figure 1:**
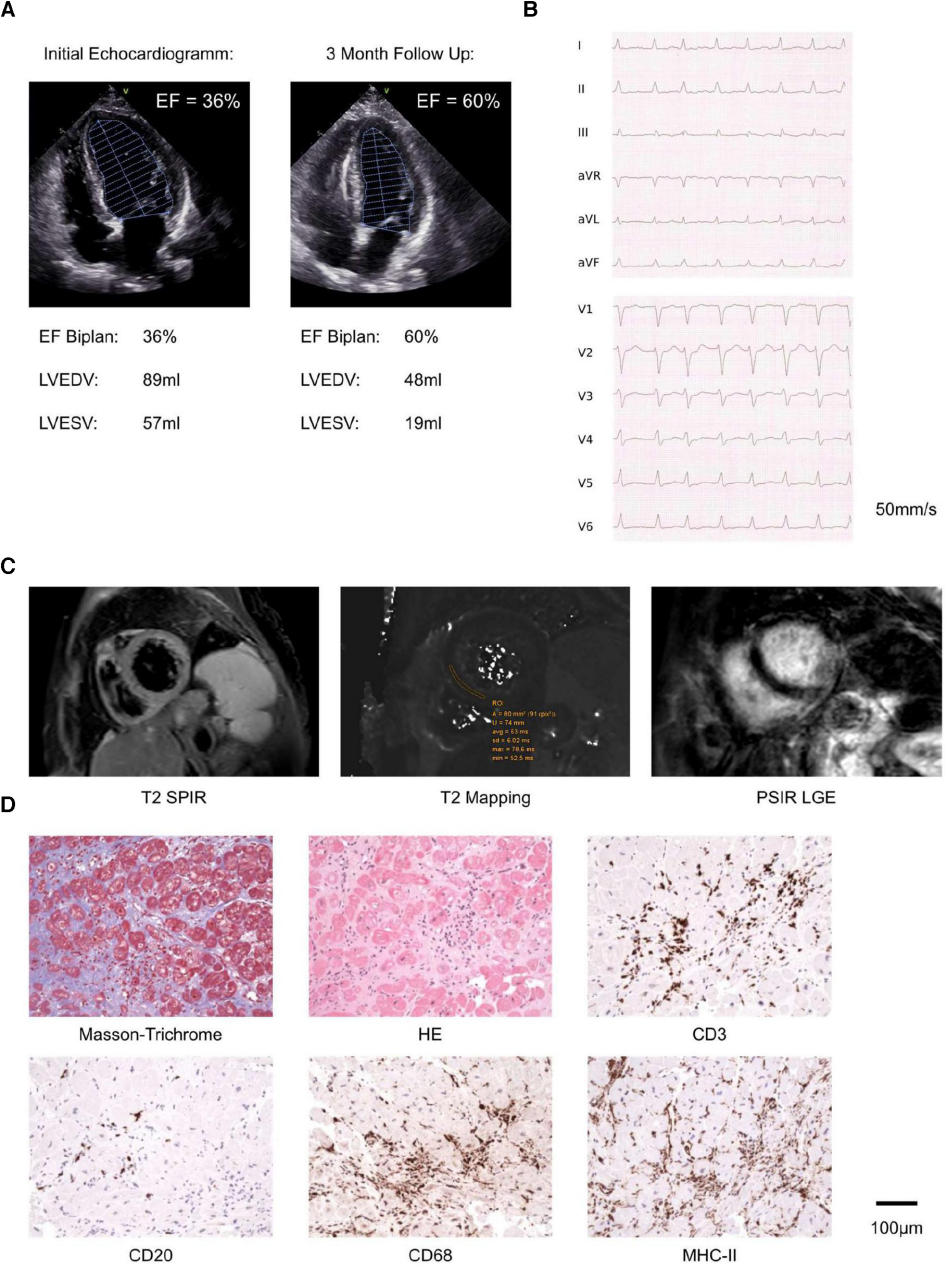
Diagnostics, including echocardiography, radiology and pathology: (**A**) representative images of initial echocardiography depicting reduced LVEF and 3 months follow up echocardiography depicting improved LVEF. (**B**) Electrocardiogram recorded during tachycardic (180/min) atrial fibrillation. (**C**) Representative images from cardiac MRI, recorded one day after initiation of prednisone. (**D**) Histopathology and Immunohistology of endomyocardial biopsies including Masson Trichrome, HE, CD3 (T lymphocytes), CD20 (B lymphocytes), CD68 (macrophages) and MHCII stains. Scale bar as indicated.

The patient was then transferred to our center and initially presented with heart failure with a reduced left ventricular ejection fraction (Figure 1A; Supplementary Report S1), which was previously unknown. Laboratory results showed elevated cardiac enzymes (Supplementary Table S1), which further increased initially, likely due to the combination of CPR and myocarditis (Supplementary Table S2). The patient was initially neuro-protectively cooled for 24 h and was successfully weaned from ventilation after 36 h. Subsequently, the patient developed fever and intermittent tachycardic atrial fibrillation, which we treated with piperacillin/tazobactam for suspected aspiration pneumonia, electrolyte supplementation, and metoprolol. Electrocardiograms exhibited no signs of ischemia, even at supraventricular tachycardic frequencies of 180 /min (Figure 1B). Hence, we postponed coronary angiography and reviewed the patient's history.

Here we learned the patient had received chemotherapy and a total of 42 infusions of 200 mg pembrolizumab at 3-weekly intervals for over 2.5 years, following the diagnosis of metastatic non-small-cell lung cancer (NSCLC, UICC stage IV). We initiated intravenous prednisone (1 mg/kg body weight) as, despite being improbable, irMyocarditis was considered a differential diagnosis. Cardiac MRI was performed the consecutive day, and MRI-based tissue characterization using Lake Louise Criteria II (LLC II) showed neither myo-/pericardial late gadolinium enhancement nor pericardial thickening or effusion. Furthermore, T2-mapping was not prolonged with an exemplary midventricular septal T2 relaxation time of 60 ms (scanner-specific reference range: 58 ± 5 ms), while native T1 relaxation was prolonged with an exemplary mid-ventricular relaxation time of 974 ms.

In summary, MRI did not indicate peri-/myocarditis (Figure 1C; Supplementary Report S2). Hence, we performed coronary catheterization and etiologically ruled out ischemia (Supplementary Report S3). We then reviewed the case within our center's interdisciplinary CIO iTox Board, dedicated to the management of irAEs, and jointly opted for endomyocardial biopsies even though imaging was inconspicuous. Five samples from the left ventricle were collected and reviewed by an external reference pathologist (KK). The myocardium showed distinct focal but also diffuse interstitial fibrosis, indicative of inflammation, likely subclinically preceding the OHCA by months. In different areas, myocyte necrosis was observed in association with numerous CD3+ *T*-cells (100 /mm^2^), a great number of CD68^+^ macrophages with MHC II expression (200 /mm^2^), and scattered CD20^+^ B-lymphocytes, without eosinophilic granulocytes or multinucleated giant cells (Figure 1D). We further carried out nested PCR to rule out cardiotropic pathogens as the underlying cause. EBV-specific DNA sequences were detected at low copy numbers in both endomyocardial biopsies and peripheral leukocyte preparation, most certainly reflecting latent systemic virus persistence. Negative PCR results were obtained for a standardized panel of further cardio-pathogens (Supplementary Table S3). Furthermore, there were no clinical signs of myasthenia or myositis and the patient could not recall pathologies of the thymus, neither did the medical history or CT scans acquired reveal any such alterations.

Taken together, (immuno)histology was consistent with acute lymphocytic myocarditis, as eosinophilic myocarditis, giant cell myocarditis, or amyloidosis were histologically excluded, thus confirming irMyocarditis. Hence, we continued prednisone treatment, on which the patient's clinical condition continuously improved to the state prior to the event. The patient received an implantable cardioverter-defibrillator for secondary prevention, pembrolizumab was permanently discontinued, and with continued heart failure medication, the patient demonstrated normalized LVEF (Figure 1A). The NSCLC remains in remission 4 months after.

## Discussion

Summarizing, while irMyocarditis resembles a comparably rare adverse event, it is associated with a high mortality of 25% to 50% (2). While previous case reports and systematic analyses on irMyocarditis exist (4, 6), the case presented herein is extraordinary in multiple aspects and thus provides compelling insights for the management of patients with (very) late-onset: Our patient experienced cardiac arrest after over 2.5 years of treatment without prior irAEs, although irMyocarditis typically manifests early after initiation (3). To our knowledge, this is the longest onset yet reported. With a rapidly growing number of patients treated with ICI, including in curative intent, this case demonstrates the importance of vigilance for irAEs even after long treatment periods. Furthermore, it stresses that early initiation of immunosuppression appears critical for improved outcomes with negligible risks if the specific event may later be attributed to a different etiology and should hence be initiated even when irMyocarditis seems unlikely (7). Moreover, it highlights that irMyocarditis may be present even with inconspicuous MRI. Although cardiac MRI became a pivotal tool in diagnosing myocarditis over the last two decades, it is critical to contemplate that while demonstrating high specificity, sensitivity is reported to be 87% with LLC II (8). Additionally, previous studies showed that sensitivity of cardiac MRI is dependent on underlying cardiac pathology and lowest in cardiomyopathic causes of myocarditis (9). Thus, especially in the context of possible irMyocarditis with inconclusive MRI, myocardial biopsies remain the gold standard of diagnosis. Additionally, our case makes evident the importance of interdisciplinary teams for optimal management of such patients. By reviewing the case in our well-established CIO iTox Board, we swiftly coordinated diagnostics and early immunosuppression, which data suggests is critical for patients’ outcomes (4, 5, 10). With the ever-growing number of ICI-based treatments and expansion beyond targeting PD-1 and CTLA-4, such interdisciplinary teams will become increasingly important in the future to manage these patients’ therapies, especially when irAEs occur. Our case also once more demonstrates, that irAEs can occur after long uneventful treatment periods, which raises the question of optimal length of ICI-based treatments. To this end, initial data indicates the possibility to achieve long-lasting remissions with time-limited treatment periods, which might reduce the risk of irAEs (11). Although mechanisms underlying early-onset and CTLA-4-mediated irMyocarditis are more and more understood and led to the implementation of abatacept as an additional treatment option (12), the biology underlying anti-PD-1 and especially late-onset irMyocarditis is unknown. Hence, further translational studies are crucial to provide optimal care to patients.

## Data Availability

The original contributions presented in the study are included in the article/Supplementary Material, further inquiries can be directed to the corresponding author.
