# Supplementary material for: Case Report: Sudden very late-onset near fatal PD1 inhibitor-associated myocarditis with out-of-hospital cardiac arrest after >2.5 years of pembrolizumab treatment
Source: Front Cardiovasc Med. 2024 Feb 19;11:1328378. doi: 10.3389/fcvm.2024.1328378 (PMC10909844; doi:10.3389/fcvm.2024.1328378)
Supplement: Supplementary file 1 [file Table1.docx]

**Supplemental Material:**

Supplemental Table 1: Full initial laboratory results

| **Parameter:** | **Value:** | **Reference range:** |
| --- | --- | --- |
| Sodium | 139 mmol/L | 135 - 145 mmol/L |
| Potassium | 4.3 mmol/L | 3.6 - 4.8 mmol/L |
| Glucose | 160 mg/dL | 74 - 109 mg/dL |
| Calcium | 2.45 mmol/L | 2.04 - 2.59 mmol/L |
| Phosphate | 2.93 mmol/L | 0.81 - 1.45 mmol/L |
| Protein | 54 g/L | 66 – 87 g/L |
| Creatinine | 1.69 mg/dL | 0.50 - 0.90 mg/dL |
| Est. GFR (CKD-EPI (2009)) | 33 mL/min |  |
| Uric acid | 10.5 mg/dL | 2.4 - 5.7 mg/dL |
| ASAT | 155 U/L | < 35 U/L |
| ALAT | 75 U/L | < 35 U/L |
| Gamma-GT | 48 U/L | < 40 U/L |
| AP | 100 U/L | 35 - 104 U/L |
| Bilirubin | 0.4 mg/dL | < 1.2 mg/dL |
| CK | 156 U/L | < 170 U/L |
| HS Troponin T | 0.098 µg/L | < 0.014 µg/L |
| LDH | 458 U/L | < 250 U/L |
| Lipase | 37 U/L | 13 - 60 U/L |
| CRP | 3.6 mg/L | < 5.0 mg/L |
| Leukocytes | 13.44 /nL | 4.40 - 11.30 /nL |
| Erythrocytes | 6.40 x10^3^ /nL | 4.0 - 5.2 x10^3^ /nL |
| Hemoglobin | 12.2 g/dL | 12.0 - 16.0 g/dL |
| Hematocrit | 40% | 36 - 45 % |
| MCV | 63 fL | 80 - 96 fL |
| MCH | 19 pg | 28 - 34 pg |
| MCHC | 30 g/dL | 31 – 37 g/dL |
| Ery. distribution width | 18.4% | 11.5 - 14.5 % |
| Platelets | 159 /nL | 150 - 400 /nL |
| TPN (Quick) | 62 % | 70 – 120 % |
| TPN (INR) | 1.2 |  |
| aPTT | 31 s | 23 - 32 |
| Derived fibrinogen | 2.32 g/L | 1.8 - 3.5 g/L |
| TSH | 9.41 mU/L | 0.27 - 4.20 mU/L |

Supplemental table 2:

| **Time point:** | **HS Troponin T:** |
| --- | --- |
| Initial presentation post CPR: | 0.098 µg/l |
| 1h after initial presentation: | 0.272 µg/l |
| 8h after initial presentation: | 0.568 µg/l |
| 1 week after initiation of immunosuppression: | 0.026 µg/l |

Supplemental table 3:

| **Viruses:** | **PCR result:** |
| --- | --- |
| Adenoviruses (ADV) | Negative |
| Coxsackieviruses of group A | Negative |
| Coxsackieviruses of group B | Negative |
| Echoviruses | Negative |
| Enteroviruses (EV) | Negative |
| Human cytomegalovirus (HCMV) | Negative |
| Human herpesvirus 6 (HHV6) | Negative |
| Human herpesvirus 7 (HHV7) | Negative |
| Herpes simplex virus type 1 (HSV1) | Negative |
| Herpes simplex virus type 2 (HSV2) | Negative |
| Parvovirus B19 (PVB19) | Negative |
| Varicella zoster virus (VZV) | Negative |
| **Bacteria:** |  |
| Borrelia spp. | Negative |
| **Parasites:** |  |
| Toxoplasma gondii | Negative |

Supplemental report 1: Echocardiography results 4 days after initiation of prednisone:

*Left Ventricle:*

Normal size, slightly concentrically hypertrophied with globally moderately impaired systolic function (Simpson Biplane 36%), no regional wall motion abnormalities, and diastolic dysfunction grade I.

*Right Ventricle:*

Normal size with normal systolic function.

*Atria:*

Left atrium slightly dilated and right atrium not dilated.

*Pericardium:*

No pericardial effusion.

*Summary:*

Left ventricle is normal in size but slightly concentrically hypertrophied with moderately impaired systolic function. The ejection fraction is 36% by Simpson biplane method. Right ventricle is normal in size with normal systolic function. The left atrium is slightly dilated.

Supplemental report 2: Full cardiac MRI report:

*Quantitative parameters of the left ventricle (LV):*

| **Parameter** | **Value** | **Reference range** |
| --- | --- | --- |
| LV end-diastolic diameter (EDD) | 54 mm  29.7 mm/m² | 36 - 55 mm  23 - 29 mm/m² |
| LV end-diastolic volume index (EDVi) | 88.2 ml/m² | 61 - 95 ml/m² |
| LV end-systolic volume index (ESVi) | 43.1 ml/m² | 17 - 35 ml/m² |
| LV ejection fraction (EF) | 51.13% | 58 - 76% |
| LV end-diastolic mass | 63.1 g/m² | 47 - 77 g/m² |
| Interventricular septum thickness | 14 mm | <10 mm |

*Quantitative parameters of the right and left atrium (end-systolic):*

| **Parameter** | **Value** | **Reference range** |
| --- | --- | --- |
| Right atrium | Area: 17.1 cm²  Area/BSA: 9.41 cm²/m² | 8 - 16 cm²/m² |
| Left atrium | Area: 14.4 cm²  Area/BSA: 7.92 cm²/m² | 8 - 16 cm²/m² |

*Tissue characterization (modified according to Lake Louise Criteria II):*

No myocardial edema. No Myocardial late gadolinium enhancement (LGE). No pericardial LGE. No pericardial thickening. No pericardial effusion.

*Myocardial mapping:*

T2-mapping: Not prolonged, exemplarily mid-ventricular septal 60 ms. T1-mapping native: Prolonged, exemplarily 974 ms mid-ventricular septal.

*Further cardiac and extracardiac findings:*

Mitral valve insufficiency. Right pleural effusion with a maximum rim width of approximately 3.2 cm. Dystelectasis in the left lower lobe of the lung. No regional motion abnormalities of the LV.

Supplemental report 3: Coronary angiography:

**
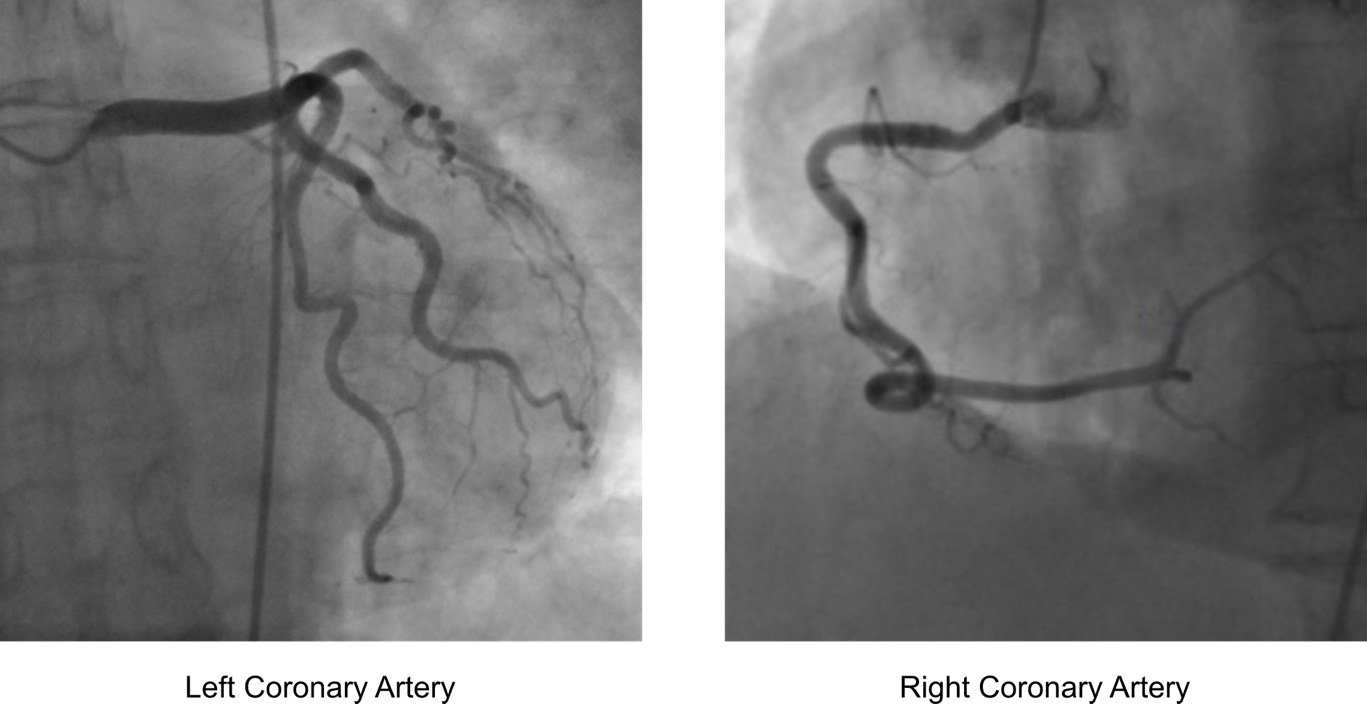
**

*Coronary angiography:*

LCA: LM, LAD and RCX without evidence of stenoses, RCA: No evidence of stenoses.
